# Caffeic Acid Phenethyl Ester Suppresses Oxidative Stress and Regulates M1/M2 Microglia Polarization via Sirt6/Nrf2 Pathway to Mitigate Cognitive Impairment in Aged Mice following Anesthesia and Surgery

**DOI:** 10.3390/antiox12030714

**Published:** 2023-03-13

**Authors:** Yue Wang, Ziwen Cai, Gaofeng Zhan, Xing Li, Shan Li, Xuan Wang, Shiyong Li, Ailin Luo

**Affiliations:** 1 Department of Anesthesiology, Hubei Key Laboratory of Geriatric Anesthesia and Perioperative Brain Health, Wuhan Clinical Research Center for Geriatric Anesthesia, Tongji Hospital, Tongji Medical College, Huazhong University of Science and Technology, 1095 Jiefang Avenue, Wuhan 430030, China; 2 Department of Cardiovascular Surgery, Union Hospital, Tongji Medical College, Huazhong University of Science and Technology, 1095 Jiefang Avenue, Wuhan 430030, China

**Keywords:** postoperative cognitive dysfunction (POCD), caffeic acid phenethyl ester (CAPE), Sirt6/Nrf2 pathway, microglia polarization, oxidative stress, neuroinflammation

## Abstract

Postoperative cognitive dysfunction (POCD) is a severe neurological complication after anesthesia and surgery. However, there is still a lack of effective clinical pharmacotherapy due to its unclear pathogenesis. Caffeic acid phenethyl ester (CAPE), which is obtained from honeybee propolis and medicinal plants, shows powerful antioxidant, anti-inflammatory, and immunomodulating properties. In this study, we aimed to evaluate whether CAPE mitigated cognitive impairment following anesthesia and surgery and its potential underlying mechanisms in aged mice. Here, isoflurane anesthesia and tibial fracture surgery were used as the POCD model, and H2O2-induced BV2 cells were established as the microglial oxidative stress model. We revealed that CAPE pretreatment suppressed oxidative stress and promoted the switch of microglia from the M1 to the M2 type in the hippocampus, thereby ameliorating cognitive impairment caused by anesthesia and surgery. Further investigation indicated that CAPE pretreatment upregulated hippocampal Sirt6/Nrf2 expression after anesthesia and surgery. Moreover, mechanistic studies in BV2 cells demonstrated that the potent effects of CAPE pretreatment on reducing ROS generation and promoting protective polarization were attenuated by a specific Sirt6 inhibitor, OSS_128167. In summary, our findings opened a promising avenue for POCD prevention through CAPE pretreatment that enhanced the Sirt6/Nrf2 pathway to suppress oxidative stress as well as favor microglia protective polarization.

## 1. Introduction

Postoperative cognitive dysfunction (POCD), a severe neurological complication after anesthesia and surgery, with a high prevalence in the elderly, prolongs hospital stays and raises disability and mortality [1,2,3,4,5]. Worldwide, the aging population grows rapidly, and as a result, the demand for effective treatments of POCD is increasing. Unfortunately, there is currently no effective clinical pharmacotherapy to prevent or cure POCD due to its unclear pathogenesis. Thus, it is essential and urgent to explore the exact mechanisms and develop effective therapeutic strategies of POCD.

Microglia, diffusely distributed throughout the brain, are involved in lots of major innate immune functions to sustain homeostasis in the central nervous system. Increasing evidence from recent studies indicated that microglia could respond to various stimuli and stress by releasing inflammatory factors and removing debris [6,7,8]. During the process of neurodegenerative diseases, microglia will be activated and dynamically change their morphology to classic (M1) or alternative (M2) activation phenotypes. Although the transformation of microglia is a continuous process and the supposed dichotomy between M1 and M2 phenotypes is now recognized as an oversimplification, this classification remains useful for understanding the function of microglia in various brain diseases [9,10,11]. Specifically, M1 phenotype microglia causes neuronal damage by releasing proinflammatory mediators. On the contrary, M2 phenotype microglia release anti-inflammatory mediators, thereby exerting beneficial effects [12,13,14]. Thus, promoting microglial activation toward the M2 phenotype appears to be a meaningful strategy for POCD treatment.

Sirtuin 6 (Sirt6) belongs to the sirtuin family of class III histone deacetylases dependent on nicotinamide adenine dinucleotide. Accumulating evidence has shown that Sirt6 participated in DNA repair, inflammation, senescence, and lipid metabolism, especially, and was also involved in aging and age-related diseases [15,16,17,18,19]. Additionally, it has been reported that the upregulation of Sirt6 could cause mice to show a drop in mature neurons and an increase in immature neurons, without disrupting glial differentiation [20]. In addition, energy restriction-induced upregulation of Sirt6 could inhibit microglial activation and enhance angiogenesis by suppressing TXNIP in cerebral ischemia [21]. Moreover, Tailin et al. reported that priming Sirt6 suppressed microglial activation, which in turn reduced LPS-induced neuroinflammation and brain ischemia injury [22]. As known, nuclear factor erythroid 2-related factor 2 (Nrf2) is a vital substrate of Sirt6, which could be activated to protect the body against oxidative damage via scavenging reactive oxygen species (ROS) [23,24]. However, whether the Sirt6/Nrf2 signaling pathway participates in the neuropathological mechanisms of POCD is still unknown.

Caffeic acid phenethyl ester (CAPE) is an activated phytochemical obtained from propolis and numerous medicinal plants. It displays vast properties, particularly antioxidation, anti-inflammation, and anti-apoptosis [25,26]. Several studies revealed a potential protective effect of CAPE for neurodegenerative diseases [27,28]. Specifically, it was revealed that CAPE alleviated cognitive impairment by modulating the activity of glycogen synthase kinase 3 beta in AD mice [29]. At present, the efficacy of CAPE in POCD mice and its potential underlying mechanisms remain to be elucidated.

Accordingly, our study aimed to evaluate the neuroprotective benefits of CAPE on aged POCD mice and its potential underlying mechanisms. We explored whether CAPE could mitigate microglia-mediated oxidative stress and favor microglia-protective polarization to alleviate POCD. Furthermore, we examined the role of the Sirt6/Nrf2 pathway induced with CAPE in combating oxidative stress and favoring microglia protective polarization in vivo and in vitro.

## 2. Materials and Methods

### 2.1. Animals

**Figure 1:**
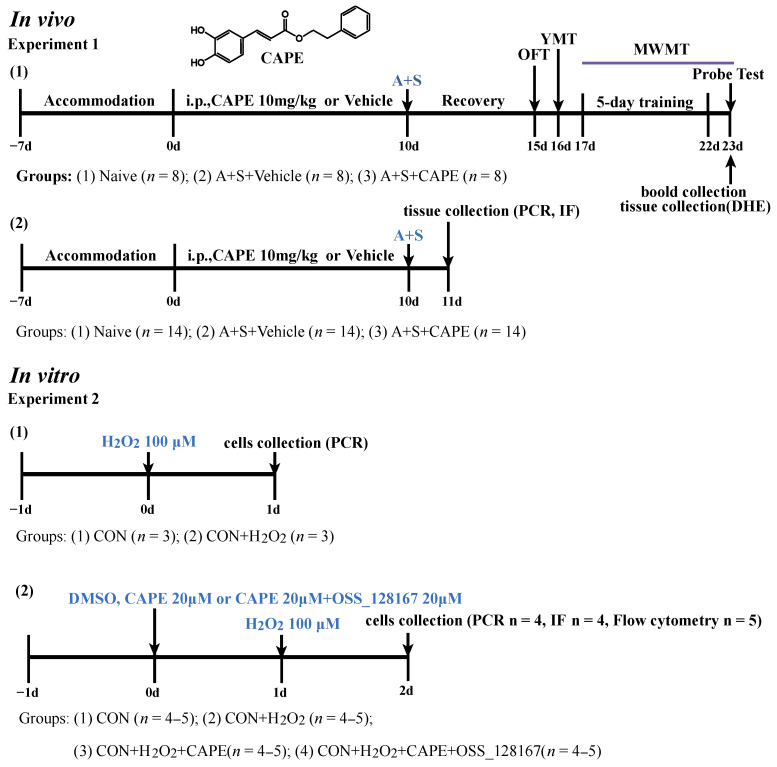
Schematic representation of the experimental procedure and grouping. Experiment 1: 20-month-old mice were pretreated with caffeic acid phenethyl ester (CAPE) or vehicle for 10 consecutive days, and then received or did not receive anesthesia and surgery. At 24 h after modeling, some of the mice were decapitated and tissue samples were collected for PCR and IF; the other mice were recovered in cages for 5 days, received behaviors assessment, and then their blood and tissue samples were collected. Experiment 2: BV2 cells were pretreated with DMSO, CAPE, or CAPE + OSS_128167 before incubating with H_2_O_2_. Subsequently, the BV2 cells were collected for PCR, IF, and flow cytometry.

A total of 66 male C57BL/6J mice (20 months old, weighing 30 to 35 g) were provided by the Laboratory Animal Center of Tongji Medical College, housed in a standard room (12 h light/dark cycle, 45–65% humidity, 22–25 °C temperature, water and food ad libitum). In our study, the mice were assigned into three groups: (1) Naive; mice received no treatment (*n* = 22); (2) A + S + Vehicle; mice only received an equivalent volume injection of solvent before anesthesia and surgery (*n* = 22); (3) A + S + CAPE (Selleck, S7414, Houston, TX, USA); mice were intraperitoneally injected with CAPE (i.p., 10 mg/kg) before anesthesia and surgery for 10 consecutive days (*n* = 22) [29] (Figure 1). CAPE was uniformly dissolved in 10% dimethyl sulfoxide, 40% PEG300, and 50% saline. The vehicle was a mixture of 10% dimethyl sulfoxide, 40% PEG300, and 50% saline. The number of animals for each group was predetermined according to numbers reported in published studies or our prior experiment, and accurate sample sizes (*n*) indicated in figure legends refer to the number of animals [30,31,32,33,34,35,36,37]. Notably, efforts were made to minimize animal suffering and the number of animals used.

### 2.2. Establishment of POCD Model

Anesthesia and surgery are usually performed in mice or rats to establish the POCD model [38,39,40,41,42,43,44,45]. Referring to published studies and our previous studies, we used isoflurane anesthesia and tibial fracture surgery as the POCD model [38,39,45,46,47]. Specifically, after one week of acclimatization, under isoflurane anesthesia, surgery, including a tibial fracture and intramedullary fixation, was performed on mice. After skin disinfection, the left tibia was revealed, fixed with a 0.3 mm pin, and then osteotomized. Afterward, a 5-0 Vicryl thread was used to suture the incision, and the incision was locally infiltrated with 0.5% bupivacaine (1 mg/kg). Subsequently, lidocaine cream was locally applied twice daily for 3 days post-surgery for incision pain. Additionally, we used a heating blanket to maintain the temperature (37 ± 0.5 °C) during the procedure. Two people completed isoflurane anesthesia and tibial fracture surgery in mice.

### 2.3. Behaviors Assessment

All of the behavioral tests were performed in a dark, quiet, and soundproof room with a comfortable temperature. All behavioral measurements were recorded using a tracking system (Zongshi Technology, Beijing, China). Each behavioral test was performed by two people who were blinded to the groups.

#### 2.3.1. Open Field Test (OFT)

The mouse was put in the neutral zone of the white chamber (50 cm × 50 cm × 50 cm) and then permitted to explore freely within 5 min. The box was sprayed with alcohol between the individual mice to eliminate odor interference. The total distance (cm) moved was measured.

#### 2.3.2. Y-Maze Test (YMT)

The YMT was carried out to assess short-term spatial working memory [48,49]. The apparatus was composed of three white acrylic arms (40 cm × 5 cm × 10 cm) separated at 120° angles. Each mouse was gently put in the distal end of an arm, and then the arm entries were recorded over 8 min. The percentage of spontaneous alternation (%SA) was calculated: %SA = [(number of alternations)/(total arm entries − 2)] × 100. Mice with less than 15 total alternations during the test were not taken into the final data.

#### 2.3.3. Morris Water Maze Test (MWMT)

The MWMT was carried out to assess long-term spatial memory function [48]. Briefly, the circular pool (diameter 1.2 m, height 50 cm) was filled with water (38-cm depth) and white tempera paint was evenly mixed to the water. A hidden white platform (diameter 10 cm) was immersed 1 cm beneath the water. The test was composed of three trials every day for 5 consecutive days and a probe test. Specifically, the mouse was gently released and permitted to search for the platform within 1 min. Mice that successfully found the platform would stay on the platform for 15 s, and mice that failed were guided to remain for the same 15 s. The time spent reaching the hidden platform (escape latency) was measured. On the sixth day, a 60-s probe trial was performed to assess reference memory, in which the platform was removed. The number of platform crossings after removing the platform was determined [50,51,52,53].

### 2.4. Reactive Oxygen Species (ROS) in the Hippocampus

The ROS levels were measured using the fluorescent probe, dihydroethidium (Beyotime, S0063, Shanghai, China). Dihydroethidium (DHE), a fluorescent probe, could be dehydrogenated with reactive oxygen species (ROS) to produce ethidium, and then ethidium could bind to RNA or DNA to produce red fluorescence. The intensity of red fluorescence is proportional to the ROS levels. Thus, the detection of red fluorescence could determine the ROS level. Following behavioral tests, mice were perfused transcardially with PBS under anesthesia, and then the brains were removed and frozen rapidly. Subsequently, the brains were sliced into 20-μm slices in a freezing microtome (Leica, CM1900, Wetzlar, Germany). Referring to published studies, frozen hippocampal sections were incubated with 5 μM dihydroethidium at 37 °C for 30 min [54,55]. Fluorescently labeled samples were imaged with a CaiZeiss confocal microscope (CaiZeiss, LSM800, Wetzlar, Germany). Specifically, the objective selected was Plan-Apochromat 20 × /0.80 Ph 2 M27, the laser power was 561 nm 0.60%, the detector gain was 643 V, and the field’s width of vision was 638.9 μm. In addition, three slides were used for analysis per mouse.

### 2.5. Oxidative Stress Indicators in Mice Plasma

Following behavioral tests, blood was collected via thoracotomy under anesthesia, and then plasma was obtained via centrifuging at 2000 rpm for 10 min. Subsequently, the plasma levels of catalase (CAT), malondialdehyde (MDA), glutathione (GSH), and superoxide dismutase (SOD) were measured using corresponding biochemical assay kits (Jiancheng Biochemical, A001-1, A006-1, A007-1, A003-1, Nanjing, China). The specific methods were performed according to the instructions.

### 2.6. Cells

BV2 cell line was applied to study microglia in our in vitro studies. BV2 cells were plated in cell culture plates (Corning Costar, 3516, Cambridge, MA, USA) and cultured with DMEM (Gibco, C119955000BT, Billings, MT, USA) containing 10% fetal bovine serum (Gibco, 10099, Billings, MT, USA). Next, the cells were firstly divided into two groups: (1) CON; cells were not treated; (2) H_2_O_2_ (Sigma, 18304, St. Louis, MO, USA); cells were treated with H_2_O_2_ (100 μM) for 24 h. Subsequently, we divided the BV2 cell populations into four groups in the further experiment: (1) CON; cells were not treated; (2) H_2_O_2_; cells were treated with H_2_O_2_ (100 μM); (3) H_2_O_2_ + CAPE; cells were pretreated with CAPE (20 μM) for 24 h and subsequently treated with H_2_O_2_ (100 μM); (4) H_2_O_2_ + CAPE + OSS_128167 (Selleck, S8627, Houston, TX, USA); cells were pretreated with CAPE (20 μM) and OSS_128167 (20 μM) [56] for 24 h, and subsequently treated with H_2_O_2_ (100 μM) (Figure 1). Sample sizes (*n*) indicated in figure legends refer to the number of biologic replicates.

### 2.7. Proliferation and Viability Assay of BV2 Cells Exposed to H_2_O_2_ and CAPE

To determine the maximum safety concentrations of H_2_O_2_ and CAPE, we performed a Cell counting kit8 (Beyotime, C0038, Shanghai, China) assay and live/dead assay (Supelco, 3106135, Bellefonte, PA, USA). Briefly, BV2 cells were seeded with three replicates per sample. When the detection time point was reached, cells were incubated with CCK8 solution for 3 h at 37 °C in 5% CO_2_ conditions after being washed. The absorbance at 450 nm was measured using a reader (Thermo Fisher, Multiskan FC, Waltham, MA, USA). For further viability detection, cells were seeded in the confocal dishes and treated with different concentrations of H_2_O_2_ or CAPE. After rinsing with DPBS, cells were incubated with Calcein-AM and PI for 30 min in the dark at 37 °C under 5% CO_2_ conditions. Images were taken with a microscope (Carl Zeiss, AXIO observer 7, Oberkochen, Germany) and analyzed using ZEN software (Carl Zeiss, Oberkochen, Germany).

### 2.8. Reactive Oxygen Species (ROS) in BV2 Cells

The ROS levels were measured with a flow cytometer (BD Biosciences, FACSCanto II, San Jose, CA, USA) using a ROS assay kit (Beyotime, S0033M, Shanghai, China). In short, cells were seeded, exposed to various concentrations of H_2_O_2_ and CAPE, and then incubated with DCFH-DA indicator for 30 min at 37 °C. Flowjo software was utilized to analyze the data.

### 2.9. Flow Cytometry (FCM)

As previously mentioned, flow cytometry was performed. In brief, firstly, cells were digested by accutase. After being washed with DPBS, a membrane-breaking fixative solution (BD Bioscience, 554714, San Jose, CA, USA) was used to fix for 15 min. Flow cytometry antibodies PE CD86 (R & D Systems, FAB741P, Minneapolis, MN, USA) and APC CD206 (R & D Systems, FAB2535A, Minneapolis, MN, USA), Rat IgG2A PE-conjugated Antibody (R & D Systems, IC006P, Minneapolis, MN, USA) and Goat IgG APC-conjugated Antibody (R & D Systems, IC108A, Minneapolis, MN, USA) were used in accordance with the instructions. Isotype controls were used in all analyses. Flow cytometry measurements were performed with FACS Calibur (BD Biosciences, FACSCanto II, San Jose, CA, USA) and analyzed using Flowjo software. All antibodies are listed in Table 1.

### 2.10. Immunofluorescence (IF)

Mice were perfused transcardially with PBS and 4% paraformaldehyde 24 h after anesthesia and surgery. Brains were excised completely, fixed with 4% paraformaldehyde, and immersed in 30% sucrose to dehydrate. Subsequently, the brains were sliced into 20-μm slices in a freezing microtome (Leica, CM1900, Wetzlar, Germany). Similarly, cells were first fixed with 4% paraformaldehyde. After being penetrated with 0.2% Triton X-100 and blocked using 5% donkey serum, the samples were incubated with appropriate primary antibodies, corresponding secondary antibodies, and DAPI successively. Fluorescently labeled samples were imaged with a CaiZeiss confocal microscope (CaiZeiss, LSM800, Oberkochen, Germany). Quantitative analysis of immunofluorescence staining images was performed by a blinded investigator using ZEN software. Specifically, we opened the original image file with ZEN software, circled Iba1+ microglia using the rectangle tool, and then obtained the average intensity of red light-labelled Sirt6 or Nrf2 using the measurement function. Additionally, three slides were used for each mouse, and ten microglia were randomly selected from each region for quantitative analysis per piece. The mean value was taken to represent the fluorescence intensity of Sirt6 or Nrf2 in the microglia. All primary and secondary antibodies are listed in Table 1.

### 2.11. Real-Time Quantitative PCR (RT-qPCR)

Total RNA was extracted from hippocampi and cells using an RNA extraction kit (FORGENE, RE-O3113, Beijing, China). Subsequently, reverse transcription was performed using HiScript III-RT SuperMix (Vazyme, R323-01, Nanjing, China). Standard RT-qPCR was performed with the ChamQ Universal SYBR qPCR Master Mix (Vazyme, Q711-02, Nanjing, China) on the Step One Plus thermal cycler (Applied Biosystems, Mississauga, ON, Canada). For the design of gene-specific primers, we first searched and selected primers sequence with validation results in Primerbank. When the primers could not be retrieved from Primerbank, the NCBI website was used for primer design, and then the designed primers were blasted to verify the specificity. Only the primers specific to the target genes were selected in this study. All primers are listed in Table 2.

### 2.12. Statistical Analysis

All data are shown as the mean ± standard error of the mean (SEM). Comparisons of results between the two groups were accessed with a *t*-test. Comparisons of results among multiple groups were accessed via one- or two-way ANOVA, followed by a Tukey post hoc test. The non-normally distributed data of platform crossing times were accessed using the Kruskal–Wallis nonparametric test and Dunnett’s post hoc test. A *p*-value below 0.05 was represented as statistically significant (* *p* < 0.05; ** *p* < 0.01; *** *p* < 0.001; **** *p* < 0.001; NS regarded as not significant). GraphPad Prism software 9.0 was used to analyze the data.

## 3. Results

### 3.1. CAPE Pretreatment Ameliorates Cognitive Dysfunction following Anesthesia and Surgery

**Figure 2:**
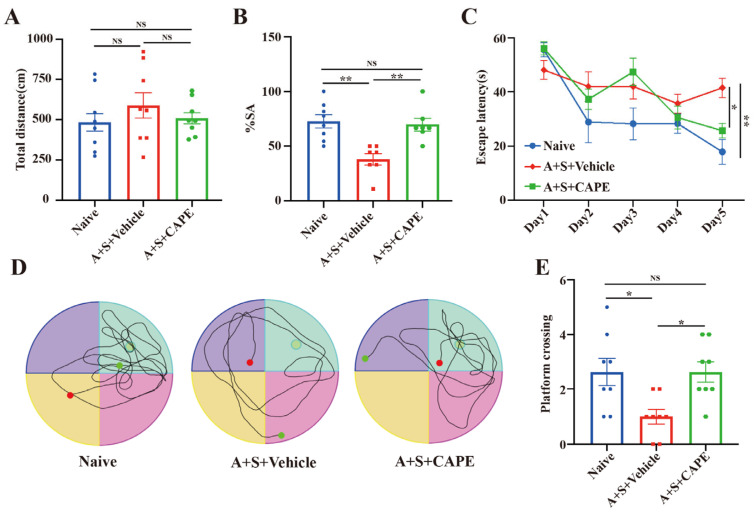
CAPE pretreatment ameliorates cognitive dysfunction following anesthesia and surgery in aged mice. (**A**) Total distance traveled in the OFT (one-way ANOVA, *p* value = 0.5207; *n* = 8 per group). (**B**) The percentage of spontaneous alternation in the YMT (one-way ANOVA, *p* value = 0.0007; Naive group *n* = 8, A + S + Vehicle group *n* = 7, A + S + CAPE group, *n* = 7). (**C**) Escape latency in five consecutive training days of the MWMT (two-way repeated measures ANOVA, time: *p* < 0.0001, groups: *p* value = 0.0460; interaction: *p* value = 0.0051; *n* = 8 per group). (**D**) Representative trace graphs on the fifth day of training in the MWMT. (**E**) Platform crossing in the probe trial of the MWMT (Kruskal–Wallis non-parametric test, *p* value = 0.0123; *n* = 8 per group). (* *p* < 0.05; ** *p* < 0.01; NS regarded as not significant).

To investigate whether CAPE pretreatment could ameliorate cognitive dysfunction following anesthesia and surgery, we randomly divided the 20-month mice into three groups: one receiving nothing, one receiving the vehicle, and another one receiving CAPE before anesthesia and surgery. Until the fifth day after anesthesia and surgery, the behavior tests were carried out (Figure 1). Firstly, OFT was performed to evaluate whether each group’s locomotor activity was consistent. The results indicated no obvious difference in the total distance (Figure 2A). Next, we performed Y-maze and MWMT to access short- and long-term spatial learning and memory function, respectively. Based on the results from the Y-maze, anesthesia and surgery reduced the percentage of spontaneous alternation behavior in aged mice, which could be abolished via CAPE pretreatment (Figure 2B). In addition, the MWMT results showed that CAPE pretreatment significantly shortened the escape latency and enhanced the number of platform crossings (Figure 2C–E). Altogether, these results demonstrated that CAPE ameliorated short- and long-term cognitive impairment following anesthesia and surgery.

### 3.2. CAPE Pretreatment Suppresses Oxidative Stress Caused by Anesthesia and Surgery

**Figure 3:**
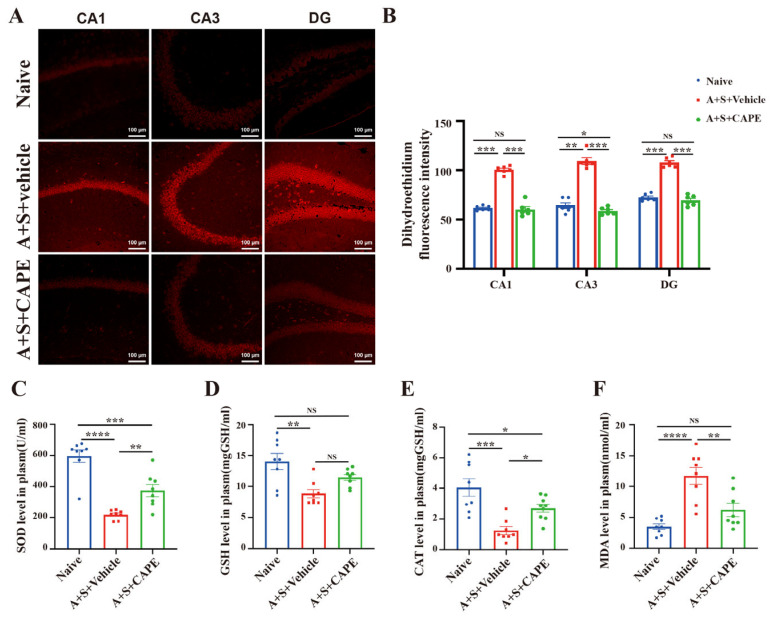
CAPE pretreatment suppresses oxidative stress caused by anesthesia and surgery in aged mice. (**A**) Representative immunofluorescence of ROS levels in the hippocampal CA1, CA3, and DG regions (*n* = 5 per group). (**B**) Quantitation of the immunofluorescent intensity for ROS levels in the hippocampal CA1, CA3, and DG regions (two-way ANOVA, Regions: *p* = 0.0010, Groups: *p* value < 0.0001, Interaction: *p* value = 0.0507; *n* = 6 per group). (**C**) The expression levels of SOD in the plasm (one-way ANOVA, *p* value < 0.0001; *n* = 8 per group). (**D**) The expression levels of GSH in the plasm (one-way ANOVA, *p* value = 0.0020; *n* = 8 per group). (**E**) The expression levels of CAT in the plasm (one-way ANOVA, *p* value = 0.0002; *n* = 8 per group). (**F**) The expression levels of MDA in the plasm (one-way ANOVA, *p* value < 0.0001; *n* = 8 per group). (* *p* < 0.05; ** *p* < 0.01; *** *p* < 0.001; **** *p* < 0.0001; NS regarded as not significant).

In view of the crucial role of oxidative stress and neuroinflammation in POCD, we sought to investigate whether CAPE pretreatment could reduce oxidative stress induced via anesthesia and surgery. Here, we used the fluorescent probe dihydroethidium to detect ROS generation in the hippocampus and revealed that anesthesia and surgery increased ROS generation. Furthermore, we found that CAPE pretreatment notably eliminated ROS generation in the hippocampal CA1, CA3, and DG regions (Figure 3A,B). In addition, we assessed the antioxidant levels, including superoxide dismutase (SOD), glutathione (GSH), and catalase (CAT), as well as the levels of fatty acids lipid peroxidation product such as malondialdehyde (MDA). The findings suggested that anesthesia and surgery triggered a marked increase in MDA, one of the most popular and reliable indicators of oxidative stress in clinical settings, and a significant diminution in SOD, GSH, and CAT in the plasm. In contrast, CAPE pretreatment dramatically increased the antioxidant (CAT and SOD) levels and reduced the MDA level in the plasm, compared with the A + S + Vehicle group (Figure 3C–E). Additionally, CAPE pretreatment raised GSH levels but was not statistically significant (Figure 3F). Collectively, the findings demonstrated that CAPE pretreatment reduced oxidative stress in the hippocampus and plasm caused by anesthesia and surgery.

### 3.3. CAPE Pretreatment Promotes the Switch of Hippocampal Microglia from the M1 to the M2 Type after Anesthesia and Surgery

**Figure 4:**
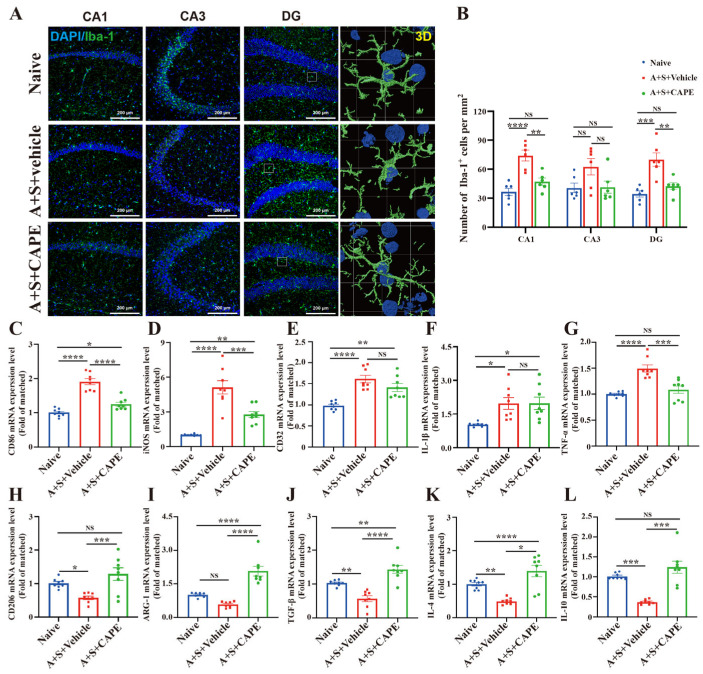
CAPE pretreatment promotes the switch of hippocampal microglia from the M1 to the M2 type after anesthesia and surgery in aged mice. (**A**) Representative immunofluorescence and 3D reconstitution images of Iba-1^+^ microglia. (**B**) The number of microglia in the CA1, CA3, and DG areas (two-way ANOVA, regions: *p* = 0.5884, groups: *p* value < 0.0001; interaction: *p* value = 0.6376; *n* = 6 per group). (**C**) RT-qPCR analysis of CD86 gene expression (one-way ANOVA, *p* value < 0.0001; *n* = 8 per group). (**D**) RT-qPCR analysis of iNOS gene expression (one-way ANOVA, *p* value < 0.0001; *n* = 8 per group). (**E**) RT-qPCR analysis of CD32 gene expression (one-way ANOVA, *p* value < 0.0001; *n* = 8 per group). (**F**) RT-qPCR analysis of IL-1β gene expression (one-way ANOVA, *p* value = 0.0071; *n* = 8 per group). (**G**) RT-qPCR analysis of TNF-α gene expression (one-way ANOVA, *p* value < 0.0001; *n* = 8 per group). (**H**) RT-qPCR analysis of CD206 gene expression (one-way ANOVA, *p* value = 0.0012; *n* = 8 per group). (**I**) RT-qPCR analysis of ARG-1 gene expression (one-way ANOVA, *p* value < 0.0001; *n* = 8 per group). (**J**) RT-qPCR analysis of TGF-β gene expression (one-way ANOVA, *p* value < 0.0001; *n* = 8 per group). (**K**) RT-qPCR analysis of IL-4 gene expression (one-way ANOVA, *p* value < 0.0001; *n* = 8 per group). (**L**) RT-qPCR analysis of IL-10 gene expression (one-way ANOVA, *p* value < 0.0001; *n* = 8 per group). (* *p* < 0.05; ** *p* < 0.01; *** *p* < 0.001; **** *p* < 0.0001; NS regarded as not significant).

Microglial polarization is known to be a response to oxidative stress and neuroinflammation. To further investigate whether CAPE could modulate M1/M2 microglia polarization in the aged POCD model, we applied confocal microscopy and three-dimensional (3D) reconstitution to analyze the morphology [57] and number of microglia in the hippocampus. As presented in Figure 4A,B, we found an obvious increase in microglial number in the hippocampal CA1 and DG regions following anesthesia and surgery, whereas the number of microglia has no significant difference in the hippocampal CA3 region. By contrast, CAPE pretreatment attenuated the alteration of microglia caused by anesthesia and surgery. Furthermore, we observed that the anesthesia and surgery-induced decrease in microglial ramification was weakened with CAPE pretreatment (Figure 4A). Based on these results, we estimated that CAPE pretreatment may facilitate the switch of hippocampal microglia from the M1 to the M2 type. To test this hypothesis, we performed RT-qPCR to evaluate the changes in microglial polarization biomarkers. As shown in Figure 4C–G, anesthesia and surgery caused an obvious elevation of M1 biomarkers (CD86, iNOS, and CD32) and pro-inflammatory cytokines (TNF-α and IL-1β). Simultaneously, the M2 biomarkers, including CD206 and TGF-β, and anti-inflammatory cytokines, including IL-4 and IL-10, were reduced after anesthesia and surgery (Figure 4H,J–L). Additionally, anesthesia and surgery reduced the level of the M2 biomarker ARG-1; however, it was not statistically significant (Figure 4I). Interestingly, CAPE pretreatment could obviously reduce the elevation of M1 biomarkers (CD86, iNOS, and CD32) and reversed the decrease in M2 biomarkers (CD206, ARG-1, and TGF-β) (Figure 4C–E,H–J). Furthermore, RT-qPCR indicated that CAPE pretreatment reversed the increased pro-inflammatory cytokines, including IL-1β and TNF-α (Figure 4F,G). Simultaneously, the decline in anti-inflammatory cytokines, including IL-4 and IL-10, was reversed with CAPE pretreatment (Figure 4K,L). Thus, our study indicated that CAPE pretreatment facilitated the switch of hippocampal microglia from the M1 to the M2 type after anesthesia and surgery.

### 3.4. CAPE Pretreatment Enhances Hippocampal Sirt6/Nrf2 Signaling Pathway following Anesthesia and Surgery

**Figure 5:**
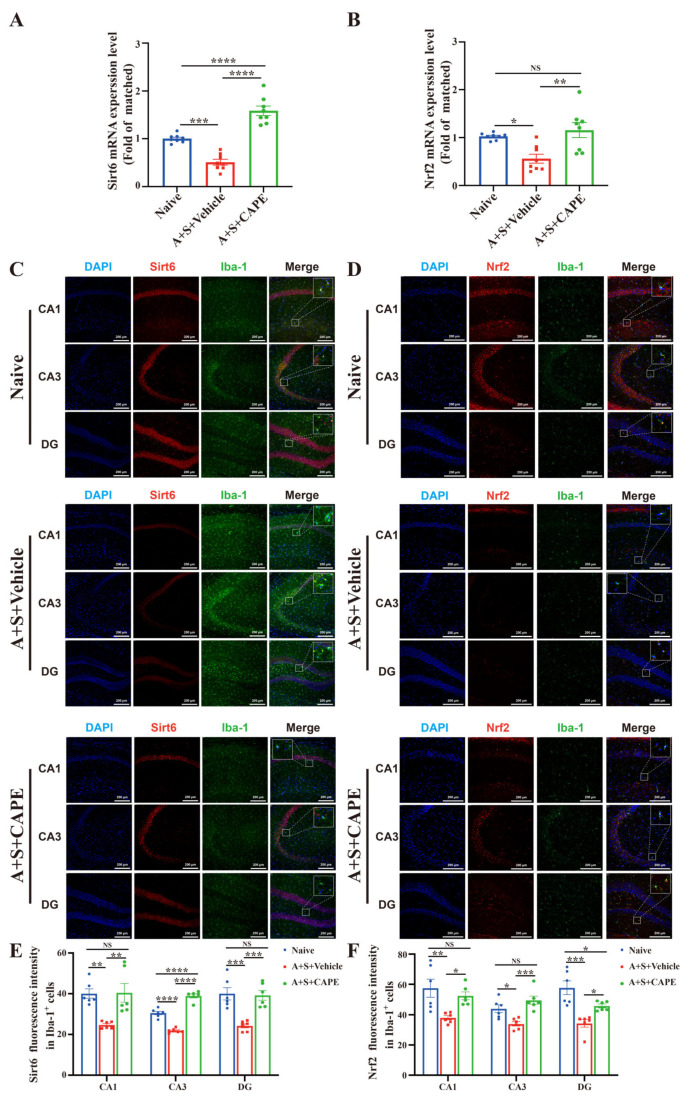
CAPE pretreatment enhances hippocampal Sirt6/Nrf2 signaling pathway after anesthesia and surgery in aged mice. (**A**) RT-qPCR analysis of Sirt6 expression in the hippocampus of aged mice among three groups (Naive, A + S + Vehicle, and A + S + CAPE) (one-way ANOVA, *p* value < 0.0001; *n* = 8 per group). (**B**) RT-qPCR analysis of Nrf2 expression in the hippocampus of aged mice among three groups (one-way ANOVA, *p* value = 0.0018; *n* = 8 per group). (**C**) Immunofluorescence staining of Sirt6 and Iba1 in the hippocampus of aged mice among three groups. (**D**) Immunofluorescence staining of Nrf2 and Iba1 in the hippocampus of aged mice among three groups. (**E**) Quantification of Sirt6 fluorescence intensity mean value in Iba-1^+^microglia among three groups (two-way ANOVA, regions: *p* = 0.0269; groups: *p* value < 0.0001; interaction: *p* value = 0.2357; *n* = 6 per group). (**F**) Quantification of Nrf2 fluorescence intensity mean value in Iba-1^+^microglia among three groups (two-way ANOVA, regions: *p* = 0.0381; groups: *p* value < 0.0001; interaction: *p* value = 0.0808; *n* = 6 per group). (* *p* < 0.05; ** *p* < 0.01; *** *p* < 0.001; **** *p* < 0.0001; NS regarded as not significant).

Sirt6 is a pivotal regulator of antioxidant response. However, whether Sirt6 contributes to POCD and its role in the efficacy of CAPE remains unclear. Here, we sought to investigate whether CAPE pretreatment could play an effective role through the Sirt6/Nrf2 pathway. Firstly, after anesthesia and surgery, we evaluated the levels of Sirt6 and Nrf2 expression in the hippocampus. The findings showed that CAPE pretreatment rescued the reduced expression levels of Sirt6 and Nrf2 caused by anesthesia and surgery (Figure 5A,B). Additionally, immunofluorescent staining analysis further demonstrated that CAPE pretreatment markedly enhanced the Sirt6 and Nrf2 expression levels of microglia in the hippocampal CA1, CA3, and DG regions following anesthesia and surgery (Figure 5C–F). Taken together, we suggested that CAPE pretreatment may mitigate cognitive dysfunction following anesthesia and surgery through enhancing the Sirt6/Nrf2 signaling pathway.

### 3.5. CAPE Alleviates H_2_O_2_-Induced ROS Generation in BV2 Cells

**Figure 6:**
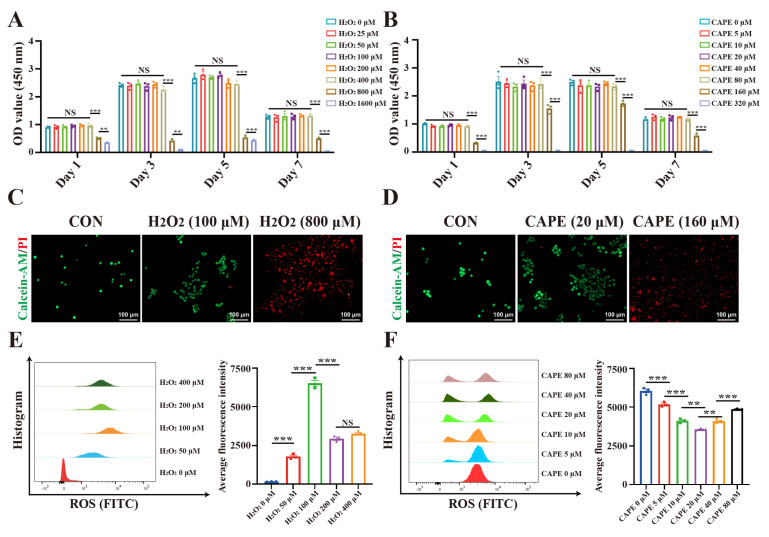
CAPE alleviates H_2_O_2_-induced ROS generation in BV2 cells. (**A**) Cell viability of BV2 cells treated with different concentrations of H_2_O_2_ at various time points (two-way ANOVA, time: *p* < 0.0001; groups: *p* value < 0.0001; interaction: *p* value < 0.0001; *n* = 3 per group). (**B**) Cell viability of BV2 cells treated with different concentrations of CAPE at various time points (two-way ANOVA, time: *p* < 0.0001; groups: *p* value < 0.0001; interaction: *p* value < 0.0001; *n* = 3 per group). (**C**) Live/dead staining of BV2 cells treated with H_2_O_2_ after 24 h (*n* = 3 per group). (**D**) Live/dead staining of BV2 cells treated with CAPE after 24 h (*n* = 3 per group). Cells were stained with Calcein-AM for live cells and with PI for dead cells. (**E**) Flow cytometry analysis of ROS levels in BV2 cells treated with different concentrations of H_2_O_2_ for 24 h (one-way ANOVA, *p* value < 0.0001; *n* = 3 per group). (**F**) Flow cytometry analysis of ROS levels in BV2 cells pretreated with different concentrations of CAPE for 24 h and then treated with 100 μM H_2_O_2_ for 24 h (one-way ANOVA, *p* value < 0.0001; *n* = 3 per group). Three independent experiments were done and for each, three replicates were plated. (** *p* < 0.01; *** *p* < 0.001; NS regarded as not significant).

To further confirm whether CAPE mitigates cognitive impairment through the Sirt6/Nrf2 signaling pathway, we performed a series of cellular experiments. Firstly, we developed H_2_O_2_-induced BV2 cells as the microglial oxidative stress model. We used the CCK-8 assays to detect the viability of BV2 cells exposed to various concentrations of H_2_O_2_ for the indicated time. These results suggested that the concentrations of H_2_O_2_ under 400 μM did not affect the viability of BV2 cells (Figure 6A). Similarly, we measured the viability of BV2 cells exposed to various concentrations of CAPE for the indicated time, demonstrating that concentrations of CAPE under 80 μM were safe for BV2 cells (Figure 6B). Live/dead staining of BV2 cells treated with H_2_O_2_ or CAPE supported the observations from the CCK-8 assays (Figure 6C,D). Since ROS is generally a reliable biomarker of oxidative stress, we applied flow cytometry to evaluate the ROS level in BV2 cells exposed to different concentrations of H_2_O_2_. The results revealed that 100 μM of H_2_O_2_ for 24 h led to the most ROS production in BV2 cells, compared with the other groups (Figure 6E). Thus, 100 μM of H_2_O_2_ for 24 h was selected to induce oxidative stress in BV2 cells. Furthermore, flow cytometry indicated the pretreatment with CAPE significantly inhibited ROS production induced by H_2_O_2_ in BV2 cells. Specifically, the H_2_O_2_-induced BV2 cells pretreated with 20 μM of CAPE show the most noticeable decrease in ROS production (Figure 6F). Altogether, CAPE pretreatment effectively alleviated H_2_O_2_-induced ROS generation in BV2 cells, and we chose 20 μM of CAPE for 24 h in subsequent experiments. 

### 3.6. CAPE Pretreatment Increases Sirt6/Nrf2 Expression Levels in H_2_O_2_-Induced BV2 Cells

**Figure 7:**
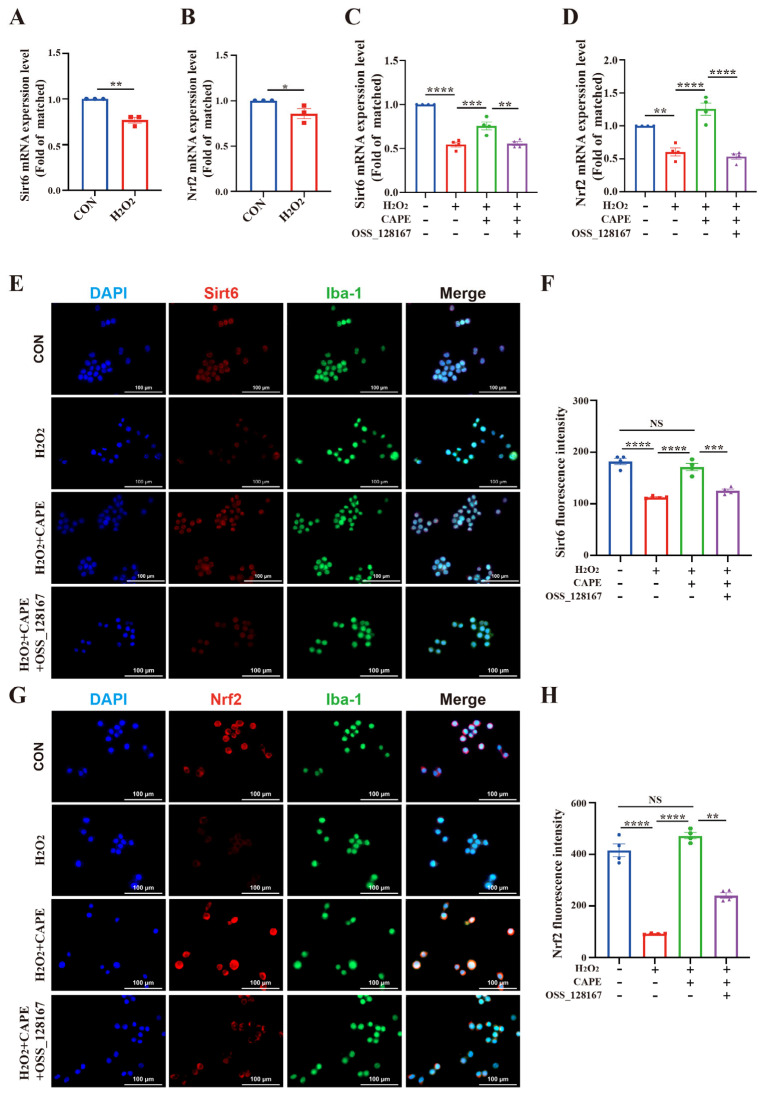
CAPE pretreatment increases Sirt6/Nrf2 expression levels in H_2_O_2_-induced BV2 cells. (**A**) RT-qPCR analysis of Sirt6 expression between the CON group and H_2_O_2_ group (*t*-test, *p* value = 0.021; *n* = 3 per group). (**B**) RT-qPCR analysis of Nrf2 expression between CON group and H_2_O_2_ group (*t*-test, *p* value = 0.0113; *n* = 3 per group). (**C**) RT-qPCR analysis of Sirt6 expression among four groups (one-way ANOVA, *p* value < 0.0001; *n* = 4 per group). (**D**) RT-qPCR analysis of Nrf2 expression among four groups (one-way ANOVA, *p* value < 0.0001; *n* = 4 per group). (**E**) Immunofluorescence staining of Sirt6 in BV2 cells after treatment with the four conditions. (**F**) Quantification of Sirt6 fluorescence intensity mean value in the four conditions (one-way ANOVA, *p* value < 0.0001; *n* = 4 per group). (**G**) Immunofluorescence staining of Nrf2 in BV2 cells after treatment with the four conditions. (**H**) Quantification of Nrf2 fluorescence intensity mean value in the four conditions (one-way ANOVA, *p* value < 0.0001; *n* = 4 per group). Three or four independent experiments were done and for each, three replicates were plated. (* *p* < 0.05; ** *p* < 0.01; *** *p* < 0.001; **** *p* < 0.0001; NS regarded as not significant).

Motivated by the observation that CAPE pretreatment enhanced hippocampal Sirt6/Nrf2 signaling pathway and suppresses oxidative stress in aged mice after anesthesia and surgery, we further explored whether the Sirt6/Nrf2 pathway plays a vital role in microglia-mediated oxidative stress. We examined Sirt6 and Nrf2 expression levels between the CON group and the H_2_O_2_ group, demonstrating that the levels of Sirt6 and Nrf2 were markedly decreased in H_2_O_2_-induced BV2 cells (Figure 7A,B). Next, we evaluated the expressions of Sirt6 and Nrf2 in H_2_O_2_-induced BV2 cells pretreated with CAPE and OSS_128167, a specific Sirt6 inhibitor. The results indicated that CAPE pretreatment markedly attenuated the decreased levels of Sirt6 and Nrf2 in BV2 cells induced by H_2_O_2_. However, this effect could be significantly inhibited by OSS_128167 (Figure 7C,D). Consistently, immunofluorescence staining supported the above results (Figure 7E–H). These results suggested the importance of the Sirt6/Nrf2 pathway in the efficacy of CAPE pretreatment in H_2_O_2_-induced BV2 cells.

### 3.7. CAPE Suppresses ROS Generation through Activating Sirt6 in H_2_O_2_-Induced BV2 Cells

**Figure 8:**
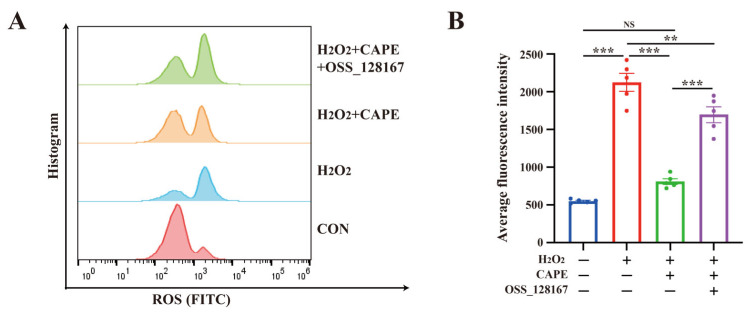
CAPE suppresses ROS generation through activating Sirt6 in H_2_O_2_-induced BV2 cells. (**A**) Flow cytometry analysis of ROS levels in BV2 cells under four conditions. (**B**) Average fluorescence intensity of BV2 cells under four conditions (one-way ANOVA, *p* value < 0.0001; *n* = 5 per group). Five independent experiments were done and for each, three replicates were plated. (** *p* < 0.01; *** *p* < 0.001; NS regarded as not significant).

The above results have demonstrated that CAPE could alleviate H_2_O_2_-induced ROS generation in BV2 cells, but whether this effect is worked via the Sirt6 pathway is still unclear. Here, we used a specific Sirt6 inhibitor, OSS_128167, to inhibit Sirt6 in H_2_O_2_-induced BV2 cells. Moreover, we applied flow cytometry to analyze whether the effect of CAPE on ROS generation would be attenuated by OSS_128167. As shown in Figure 8A,B, OSS_128167 obviously weakened the inhibitory effect of CAPE on ROS generation. Therefore, we suggested that CAPE suppressed ROS generation through activating Sirt6 in H_2_O_2_-induced BV2 cells.

### 3.8. CAPE Promotes the Switch of H_2_O_2_-Induced BV2 Cells from the M1 to the M2 Type through Activating Sirt6

**Figure 9:**
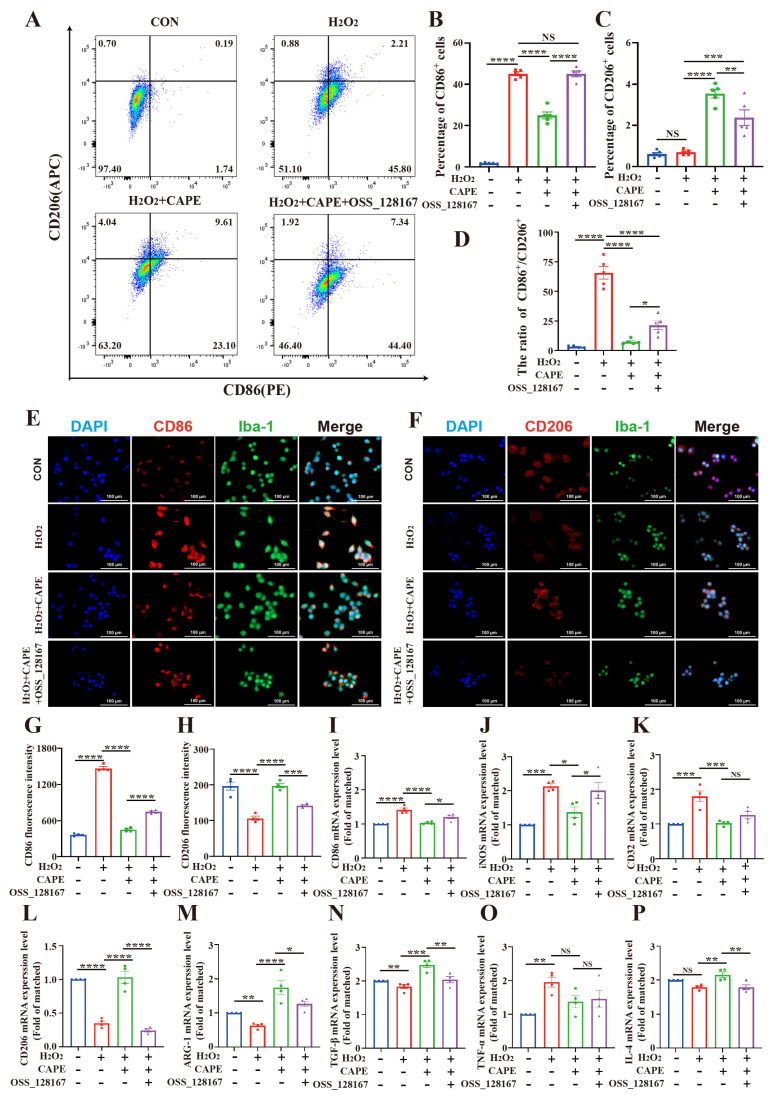
CAPE promotes the switch of H_2_O_2_-induced BV2 cells from the M1 to the M2 type through activating Sirt6. (**A**) Representative flow cytometry analysis of BV2 cells induced by four conditions. (**B**) Quantification of the percentage of CD86^+^ cells after treatment with four conditions (one-way ANOVA, *p* value < 0.0001; *n* = 5 per group). (**C**) Quantification of the percentage of CD206^+^ cells after treatment with four conditions (one-way ANOVA, *p* value < 0.0001; *n* = 5 per group). (**D**) The ratio of CD86^+^/CD206^+^ cells after treatment with four conditions (one-way ANOVA, *p* value < 0.0001; *n* = 5 per group). (**E**) Immunofluorescence staining of CD86 and Iba1 in BV2 cells after treatment with the four conditions. (**F**) Immunofluorescence staining of CD206 and Iba1 in BV2 cells after treatment with the four conditions. (**G**) Quantification of CD86 fluorescence intensity mean value in the four conditions (one-way ANOVA, *p* value < 0.0001; *n* = 4 per group). (**H**) Quantification of CD206 fluorescence intensity mean value in the four conditions (one-way ANOVA, *p* value < 0.0001; *n* = 4 per group). (**I**) RT-qPCR analysis of CD86 in BV2 cells under four conditions (one-way ANOVA, *p* value < 0.0001; *n* = 4 per group). (**J**) RT-qPCR analysis of iNOS in BV2 cells under four conditions (one-way ANOVA, *p* value = 0.0004; *n* = 4 per group). (**K**) RT-qPCR analysis of CD32 in BV2 cells under four conditions (one-way ANOVA, *p* value = 0.0002; *n* = 4 per group). (**L**) RT-qPCR analysis of CD206 in BV2 cells under four conditions (one-way ANOVA, *p* value < 0.0001; *n* = 4 per group). (**M**) RT-qPCR analysis of ARG-1 in BV2 cells under four conditions (one-way ANOVA, *p* value = 0.0001; *n* = 4 per group). (**N**) RT-qPCR analysis of TGF-β in BV2 cells under four conditions (one-way ANOVA, *p* value = 0.0002; *n* = 4 per group). (**O**) RT-qPCR analysis of the pro-inflammatory cytokine TNF-α in BV2 cells under four conditions (one-way ANOVA, *p* value = 0.0161; *n* = 4 per group). (**P**) RT-qPCR analysis of the anti-inflammatory cytokine IL-4 in BV2 cells under four conditions (one-way ANOVA, *p* value = 0.0018; *n* = 4 per group). Five or four independent experiments were done and for each, three replicates were plated. (* *p* < 0.05; ** *p* < 0.01; *** *p* < 0.001; **** *p* < 0.0001; NS regarded as not significant).

To further confirm whether CAPE promotes the switch of microglia from the M1 to the M2 type through Sirt6/Nrf2 pathway in H_2_O_2_-induced BV2 cells, we evaluated the status of microglia among the CON, H_2_O_2_, H_2_O_2_ + CAPE, and H_2_O_2_ + CAPE + OSS_128167 groups. Flow cytometry analyses revealed a strong increase in M1-type microglia (CD86^+^) in H_2_O_2_-induced BV2 cells, with no significant difference regarding M2 ones (CD206^+^). Furthermore, pretreatment with CAPE promoted the switch of microglia from the M1 to M2 type. However, this effect was weakened by OSS_128167 (Figure 9A–D). Then, immunofluorescence accessed the expressions of CD86 and CD206. As presented in Figure 9E–H, CAPE pretreatment could decrease CD86 expression and enhance CD206 expression in BV2 cells following H_2_O_2_ exposure. In addition, RT-qPCR confirmed that pretreatment with CAPE reduced the expression of M1 biomarkers (CD86, iNOS, and CD32) (Figure 9I–K) and increased the expression of M2 biomarkers (CD206, Arg-1, and TGF-β) (Figure 9L–N) in the H_2_O_2_-induced BV2 cells, which also could be suppressed by OSS_128167. We further demonstrated that CAPE decreased the expression of TNF-α (Figure 9O) and elevated the expression of IL-4 (Figure 9P). Thus, these findings indicated that CAPE promoted the switch of H_2_O_2_-induced BV2 cells from the M1 to M2 type through the Sirt6 pathway.

## 4. Discussion

Acquiring an effective prophylactic medication is crucial to the treatment of POCD. Currently, there is no effective clinical pharmacotherapy to prevent POCD. CAPE, a natural constituent of propolis and numerous medicinal plants, shows powerful biological properties. A previous study has revealed that CAPE ameliorated cognitive dysfunction and dementia in AD mice by upregulating the Nrf2/HO-1 pathway [29]. In addition, accumulating studies showed that CAPE could upregulate the PI3-kinase-dependent pathway and downregulate the JAK/STAT pathway, thereby ameliorating cognitive impairment caused by drug toxicity [58,59]. Therefore, we further explored its role in the aged POCD model and expanded the application scope of CAPE. In the present study, we revealed that CAPE pretreatment could ameliorate short- and long-term spatial learning and memory impairment after anesthesia and surgery in aged mice. More importantly, our mechanistic studies revealed that CAPE pretreatment could suppress oxidative stress and facilitate the switch of microglia from the M1 to M2 type by enhancing the Sirt6/Nrf2 pathway in the hippocampus to ameliorate cognitive dysfunction after anesthesia and surgery. Thus, CAPE pretreatment may be a meaningful therapeutic strategy for POCD prevention in the future.

Oxidative stress and prolonged neuroinflammation have been considered the main pathological factors contributing to POCD development [39,60,61,62]. A few studies have suggested that anesthesia and surgery resulted in the increase in malondialdehyde and oxidative damage in the elderly brain and antioxidants could attenuate cognitive dysfunction after anesthesia and surgery [45,63,64,65]. Moreover, emerging evidence suggested that suppression of neuroinflammation could attenuate cognitive deficits caused by laparotomy and cardiopulmonary bypass surgery under isoflurane anesthesia [66,67]. In this study, we found that CAPE pretreatment notably eliminated ROS generation in the hippocampus and reversed the elevation of catalase (CAT) and the decrease in glutathione (GSH) and superoxide dismutase (SOD) in the plasma, thereby ameliorating cognitive dysfunction following anesthesia and surgery.

Mounting studies reported that microglia are merged as a critical gatekeeper of brain homeostasis, which exerts its regulatory effects in oxidative stress and neuroinflammation [68,69,70]. Microglial phenotypes could influence disease progression in the brain through their balance between M1 and M2-activated states [71]. Thus, we focused on the alternation of microglia and its related molecular mechanisms in aged mice undergoing anesthesia and surgery. Previous studies from our team and other laboratories indicated that anesthesia and surgery led to a notable increase in microglial activation in the hippocampi of rats [30,61,72]. Here, we demonstrated that CAPE pretreatment facilitated the switch of microglia from the M1 to M2 type in the hippocampi of aged mice to ameliorate cognitive dysfunction following anesthesia and surgery. However, the underlying molecular mechanism of microglial polarization still warrants exploration.

Sirt6 is a deacetylase and plays a vital role in inhibiting oxidative stress and driving macrophage polarization toward the M2 type [73,74]. Specifically, it has been reported that the upregulation of the Sirt6/Nrf2 pathway ameliorated alcoholic liver disease and APAP-induced hepatotoxicity via protecting against oxidative stress [74,75]. Additionally, Song et al. demonstrated that adipose Sirt6 maintained systemic insulin sensitivity by deriving macrophage polarization toward M2 [73]. Moreover, recent studies showed that Sirt6 overexpression inhibited the inflammatory response and thus ameliorate neurological deficits in intracerebral hemorrhage rats and cerebral ischemia and reperfusion rats [21,76]. Additionally, endothelial Sirt6 could exert a meaningful effect in ischemic strokes by guarding blood–brain barrier (BBB) integrity [77]. Based on our results that CAPE pretreatment reduced oxidative stress and facilitated the switch of microglia from M1 to M2 polarization in the hippocampi of aged mice after anesthesia and surgery, we further evaluated the vital role of the Sirt6/Nrf2 pathway in these effects. As expected, we found that CAPE pretreatment could reduce ROS production and promote the expression of M2 phenotype markers via enhancing the Sirt6/Nrf2 pathway in vivo and in vitro, however, the effects of CAPE would be obviously weakened by the specific Sirt6 inhibitor, OSS_128167, in H_2_O_2_-induced BV2 cells. Therefore, we suggested that CAPE pretreatment decreased oxidative stress and favored microglia transforming toward the M2 type via activating the Sirt6/Nrf2 signaling pathway, thereby ameliorating cognitive impairment.

Actually, there are still many issues awaiting further investigations in the future. Firstly, although we demonstrated that CAPE pretreatment could ameliorate cognitive dysfunction following anesthesia and surgery through facilitating the transformation of microglia toward M2 type via the Sirt6/Nrf2 signaling pathway, other cells besides microglia may also contribute to contributing to the effects. The effect of the Sirt6/Nrf2 pathway in neurons and astrocytes still needs to be investigated. Secondly, we mainly focused on the changes in Sirt6 expression and microglial polarization in the early stage of POCD. It is of great significance to further explore the related molecular changes in the late stage. Additionally, due to the complexity of the in vivo environment and technological restrictions, it is difficult to match the in vitro concentration of CAPE with the in vivo dose. Furthermore, we did not explore the differential effect of surgical procedures and anesthesia on the expression of Sirt6, as we used the model to mimic the clinical settings where it is rare to separate the two steps. However, the effect of surgical procedures and anesthesia on oxidative stress and neuroinflammatory response is variable [78,79]. Moreover, a published study has reported that CAPE could reverse cadmium-induced cognitive impairment in mice through the AMPK/Sirt1 pathway [80]. Furthermore, our previous study has found that resveratrol could alleviate cognitive impairment by activating Sirt1 in aged rats after anesthesia and surgery [31]. Thus, it is worth exploring whether CAPE also mediated the Sirt1 pathway to alleviate cognitive impairment in elderly mice after anesthesia and surgery.

## 5. Conclusions

In summary, we found that CAPE pretreatment alleviated cognitive impairment in aged mice following anesthesia and surgery. Moreover, CAPE pretreatment upregulated the Sirt6/Nrf2 pathway, attenuated oxidative stress, and favored microglia transforming toward the M2 type in vivo and in vitro. These findings suggested that CAPE pretreatment may be a promising therapeutic strategy for POCD prevention through enhancing the Sirt6/Nrf2 pathway to suppress oxidative stress as well as favor microglia protective polarization.

## Figures and Tables

**Table 1:** All primary and secondary antibodies used in this study.

Antibody	Species	IF	FCM	Source	Catalogue
Nrf2	Rabbit	1:200		Proteintech	16396-1-AP
Sirt6	Rabbit	1:300		Proteintech	13572-1-AP
Iba-1	Goat	1:100		Abcam	ab5067
CD86	Rabbit	1:100		Abclonal	A16805
CD206	Rabbit	1:100		Proteintech	18704-1-AP
CoraLite594-conjugated Donkey Anti-Rabbit lgG (H + L)		1:100		Proteintech	SA00013-8
FITC-conjugated Affinipure Donkey Anti-Goat lgG (H + L)		1:50		Proteintech	SA00003-3
Mouse B7-2/CD86 PE-conjugated Antibody	Rat		1:20	R & D Systems	FAB741P
Mouse MMR/CD206 APC-conjugated Antibody	Goat		1:20	R & D Systems	FAB2535A
Rat IgG2A PE-conjugated Antibody	Rat		1:20	R & D Systems	IC006P
Goat IgG APC-conjugated Antibody	Goat		1:20	R & D Systems	IC108A

**Table 2:** Primers used in this study.

Primer Name	Primer Sequences (5′-3′)	
	Forward	Reverse
Sirt6	CTCCAGCGTGGTTTTCCACA	GCCCATGCGTTCTAGCTGA
Nrf2	CTGAACTCCTGGACGGGACTA	CGGTGGGTCTCCGTAAATGG
CD86	GGTGGCCTTTTTGACACTCTC	TGAGGTAGAGGTAGGAGGATCTT
CD206	GCTTCCGTCACCCTGTATGC	TCATCCGTGGTTCCATAGACC
iNOS	CAAGCACCTTGGAAGAGGAG	AAGGCCAAACACAGCATACC
CD32	GGAATCCTGCCGTTCCTACTG	ATGGCACAAAGTCCGTGAGAA
ARG1	TGTCCCTAATGACAGCTCCTT	GCATCCACCCAAATGACACAT
TGFβ1	CCACCTGCAAGACCATCGAC	CTGGCGAGCCTTAGTTTGGAC
TNF-α	CAGGCGGTGCCTATGTCTC	CGATCACCCCGAAGTTCAGTAG
IL-4	GGTCTCAACCCCCAGCTAGT	GCCGATGATCTCTCTCAAGTGAT
IL-1β	TTCAGGCAGGCAGTATCACTC	GAAGGTCCACGGGAAAGACAC
IL-10	AGCCTTATCGGAAATGATCCAGT	GGCCTTGTAGACACCTTGGT
GAPDH	AGTGCCAGCCTCGTCCCGTAGACAA	CAGGCGCCCAATACGGCCAAAT

## Data Availability

The data used in this study are available upon request.
